# Factors Associated with Discharge to a Skilled Nursing Facility after Transcatheter Aortic Valve Replacement Surgery

**DOI:** 10.3390/ijerph16010073

**Published:** 2018-12-28

**Authors:** Carolyn E. Horne, Tamara S. Goda, L. Wiley Nifong, Alan P. Kypson, Wesley T. O’Neal, Linda C. Kindell, Charulata Jindal, Jimmy T. Efird

**Affiliations:** 1 College of Nursing, East Carolina University, Greenville, NC 27858, USA; hornec@ecu.edu; 2 Cardiovascular Sciences, East Carolina Heart Institute at Vidant Medical Center Greenville, Greenville, NC 27858, USA; tammy.goda@vidanthealth.com; 3 Robotic Surgery Center for Training and Education, East Carolina Heart Institute, Greenville, NC 27858, USA; nifongl@ecu.edu; 4 Cardiac Surgical Specialists, REX Health Care, University of North Carolina, Raleigh, NC 27858, USA; alan.kypson@unchealth.unc.edu; 5 Division of Cardiology, Emory University, Atlanta, GA 30322, USA; wesley.oneal@emory.edu; 6 Department of Cardiovascular Sciences, Brody School of Medicine, East Carolina University, Greenville, NC 27858, USA; kindelll@ecu.edu; 7 Centre for Clinical Epidemiology and Biostatistics (CCEB), The University of Newcastle (UoN), Newcastle 2308, Australia; charu.jindal@uon.edu.au; 8 Research Centre for Generational Health and Aging (RCGHA), The University of Newcastle (UoN), Newcastle 2308, Australia

**Keywords:** aortic stenosis, risk stratification, skilled nursing facility, transcatheter aortic valve replacement

## Abstract

An assumption regarding transcatheter aortic valve replacement (TAVR), a minimally invasive procedure for treating aortic stenosis, is that patients remain at, or near baseline and soon return to their presurgical home to resume activities of daily living. However, this does not consistently occur. The purpose of this study was to identify preoperative factors that optimally predict discharge to a skilled nursing facility (SNF) after TAVR. Delineation of these conditions is an important step in developing a risk stratification model to assist in making informed decisions. Data was extracted from the American College of Cardiology (ACC) transcatheter valve therapy (TVT) registry and the Society of Thoracic Surgeons (STS) database on 285 patients discharged from 2012–2017 at a tertiary referral heart institute located in the southeastern region of the United States. An analysis of assessment, clinical and demographic variables was used to estimate relative risk (RR) of discharge to a SNF. The majority of participants were female (55%) and white (84%), with a median age of 82 years (interquartile range = 9). Approximately 27% (*n* = 77) were discharged to a SNF. Age > 75 years (RR = 2.3, *p =* 0.0026), female (RR = 1.6, *p* = 0.019), 5-meter walk test (5MWT) >7 s (RR = 2.0, *p* = 0.0002) and not using home oxygen (RR = 2.9, *p* = 0.0084) were identified as independent predictive factors for discharge to a SNF. We report a parsimonious risk-stratification model that estimates the probability of being discharged to a SNF following TAVR. Our findings will facilitate making informed treatment decisions regarding this older patient population.

## 1. Introduction

By the year 2030, 72 million people will be over the age of 65 years [1]. A common cardiac dysfunction among older patients is aortic stenosis (AS) caused by calcification of a normal trileaflet aortic valve [2]. The occurrence of AS ranges from 9% among younger patients with a mean age of 54 years, compared with 42% among older patients with a mean age of 81 years [3].

Transcatheter aortic valve replacement (TAVR) is a minimally invasive surgical procedure that has been widely adopted throughout the United States (U.S.) and Europe as an acceptable alternative to traditional surgery for aortic valve replacement of high-risk patients [4,5]. Compared with standard therapy, transfemoral TAVR has been associated with lower mortality and a reduced rate of repeat hospitalization [6]. This procedure also may be the only option for older patients with AS who are deemed ineligible or at high risk for traditional surgical aortic valve replacement (SAVR).

The ideal outcome for a patient undergoing TAVR is successful implantation with discharge after a one- to two-day hospitalization, returning to their presurgical home and resuming normal activities of daily living. Although home discharge has been shown to be associated with improved survival and less resource utilization following valve surgery, TAVR patients frequently are discharged to a skilled nursing facility (SNF) for rehabilitation [7,8,9,10,11,12,13,14,15,16]. Even with the additional services available at a SNF, the majority of patients do not regain sufficient functionality to enable their eventual return to the community [17]. Understanding the dynamics of this process can aid in assessing the risk of patients who may or may not be suitable for this procedure. 

The purpose of this study was to identify a parsimonious set of preoperative characteristics, which were independently associated with discharged to a SNF following TAVR. The use of these factors in a risk-stratification model is an important decision making tool to help decrease SNF admission rates. 

## 2. Methodology

### 2.1. Population and Study Design

The study population consisted of patients receiving TAVR between January 2012 and March 2017 at a large tertiary cardiac hospital in the southeastern region of the U.S. Patients predominantly underwent TAVR by way of a transfemoral access site, compared with other more technically difficult approaches (e.g., transapical, transthoracic). Assessment, clinical, and demographics variables were obtained from the prospectively maintained American College of Cardiology (ACC) transcatheter valve therapy (TVT) registry and the Society of Thoracic Surgeons (STS) database, after obtaining institutional review board (IRB) approval (UMCIRB 17-000708). Discharge from hospitalization on the same day or subsequent day to a SNF was the outcome variable of interest. Following a review of the literature, independent (preoperative) variables were considered in our analyses if they were potentially associated with discharge to a SNF.

### 2.2. Definition of a Skilled Nursing Facility

A SNF was defined as a separate facility or a special unit of a hospital that provides medically necessary inpatient nursing care around the clock, under the supervision of a registered nurse. Such a facility typically employs or contracts skilled rehabilitation specialists (e.g., physical and occupational therapists, speech pathologists and audiologists) to help restore physical function and abilities, under the expectation of partial or complete restoration of the latter. 

### 2.3. Statistical Analysis

Comparisons of variables with respect to being discharged to a SNF were made using Fisher’s exact test for categorical variables and the Deuchler-Wilcoxon procedure for continuous variables. A multivariable log-binomial (regression) analysis of assessment, clinical, and demographic variables was used to estimate relative risk (RR) of discharge to a SNF and to construct a risk-stratification matrix [18]. *p*-values were computed using a likelihood ratio procedure. The arcsine (angular transformation) method was used to compute 95% confidence intervals for the estimated probability of discharge to a SNF [19]. Preoperative characteristics were included in the multivariable model if *p* < 0.025 and RR > 1.5 (or <0.67) in univariable analysis. This systematic variable selection method leads to better model reliability and stability. The Akaike information criterion (AIC) and c-statistic were used to assess model quality and discrimination. All models satisfied convergence and admissibility criteria (i.e., linear predictor constrained to be negative) [20]. A multistage expectation-maximization (EM) algorithm was used to handle missing values [21]. The method established by Holly and Whittemore was used for rounding [22]. Statistical analyses were performed using SAS software (version 9.4, SAS Inc., Cary, NC, USA).

## 3. Results

A total of 285 patients underwent TAVR, of whom 84% were white (Table 1). The median age was 82 years (interquartile range = 9). Approximately 27% (*n* = 77) were discharged to a SNF. Important differences by discharge status were observed for age, sex, 5-meter walk test (5MWT) and home oxygen use prior to surgery (meeting the a priori criteria of *p* < 0.025 and RR > 1.5 (or RR < 0.67)).

On multivariable analysis, age > 75 years, 5MWT > 7 s and negative home oxygen use were associated with a ≥ 2-fold adjusted RR for discharge to a SNF following TAVR (*p* ≤ 0.0084) (Table 2). Additionally, females were 60% more likely than men to be discharged to a SNF (*p* = 0.019). 

The greatest probability of discharge to a SNF corresponded to female patients >75 years of age who required >7 s to walk 5 m and were not suitable for home oxygen use (59%, 95% CI = 54%–65%) (Table 3). In comparison, the lowest discharge probability corresponded to male patients ≤75 years of age who required ≤7 s to walk 5 m and used home oxygen prior to procedure (3%, 95% CI = 1%–5%). Our multivariable model was well-calibrated and provided moderate discrimination beyond chance, with a low AIC value of 300. 

## 4. Discussion

First performed in 2002, TAVR provides an alternative treatment option for patients who are unable to tolerate a surgical procedure for AS [23]. The procedure yields improved functionality, better quality of life, and lower cost compared with SAVR and medical therapy [7]. Owing to the less invasive nature of this method, TAVR also is associated with fewer bleeding episodes, less chances of acute kidney injury, a lower frequency of new-onset atrial fibrillation, and shorter intensive care unit (ICU)/hospital length of stay (LOS) [24]. 

Patients with symptomatic AS typically are referred for TAVR because of advanced age, multiple comorbidities, previous cardiovascular procedures, and a decreased life expectancy [25]. Approximately 30%–40% of patients are denied traditional surgical repair of the aortic valve. If AS is left untreated in those who are non-surgical candidates, the mortality rate for severe symptomatic AS is ~50% within one year of diagnosis [6,26].

While the majority of outcomes following TAVR have been positive, there continues to be concern of patients requiring disposition to a SNF and to remain there for an extended period. An assumption regarding TAVR is that patients remain at, or near baseline given the minimally invasive nature of the procedure; expecting most patients to return home and resume preoperative activities of daily living. Often this is not the case and highlights the need to identify preoperative factors that are helpful in predicting which patients will be discharged to a SNF following TAVR.

In our study, we examined the ACC TVT registry and STS database to identify preoperative characteristics independently associated with discharge status among patients who underwent TAVR at our institution. This information, in the form of a risk stratification matrix, was then used to estimate a patient’s probability of discharge to a SNF, given their risk profile. Our results suggest that there are specific preoperative characteristics that independently predict which patients are discharged to a SNF. For, example, older women with prolonged 5MWT scores and no home oxygen use prior to procedure were at highest risk for discharge to a SNF, compared with other risk factors profiles.

### 4.1. Importance of Discharge Disposition

In recent years, the emphasis to reduce costs and nosocomial infections following cardiovascular surgery has led to correspondingly shorter hospitalization times and a greater need to better understand characteristics associated with a patient’s discharge disposition [27]. Such knowledge ultimately will help to predict which patients will be discharged to a SNF. Preoperative characteristics potentially important in this decision-making process include patient assessment, clinical, and demographics characteristics [12,27,28,29,30].

Discharge to a SNF has been associated with increased mortality and 30-day readmission rates (in some but not all studies) [12,31]. In contrast, patients who are not discharged to a SNF often regain ambulation sooner when in their known environment and have a reduced occurrence of infection. Other benefits of the latter include shorter healing times and increased quality of life. 

### 4.2. Review of Discharge Literature 

Discharge to a SNF accounts for a significant proportion of Medicare costs, with benefits covering stays up to 100 days for patients who require specialized care [32]. To the best of our knowledge, no study has examined the factors associated with discharge to a SNF following TAVR. However, such factors has been elucidated for other acute hospitalizations such as heart failure. 

In an analysis of Medicare beneficiaries ≥65 years of age, hospitalized for heart failure, longer LOS, advanced age, being female, hypotension, higher ejection fraction, absence of ischemic heart disease, and various comorbidities were associated with discharge to a SNF [28]. Among this group, 27% were rehospitalized within 30 days and over half died within one year. Consistent with this study, we observed an increased risk of discharge to a SNF among older female TAVR patients. 

The likelihood of patients with heart failure being admitted to hospitals with lower SNF rates has been reported to be higher among black and other priority populations. A significant ethnic disparity also has been observed for Hispanic patients hospitalized for trauma, with this group having the lowest adjusted odds of being discharged to a SNF (OR = 0.55, 95% CI = 0.53–0.58) [33]. In our analysis, race was not a significant predictor of discharge status following TAVR [34].

In a comparison of patients with end stage renal disease who underwent SAVR in the National Inpatient Sample (NIS) and National Readmission Database (NRD), those receiving TAVR were less likely to be discharged to a SNF (43% vs. 56%; *p* = 0.01) [35]. Approximately 27% of patients undergoing surgery at our dedicated heart institute were discharged to a SNF following TAVR.

Among patients undergoing TAVR from late 2011 through the middle of 2013 at 299 US-based hospitals, 33% were discharged to a skilled transitional care unit [36]. In contrast to our study, this lower number likely reflects differences in surgical approach, with few patients in our study undergoing TAVR by way of a transapical access site. However, no information was provided in the above study regarding risk factors for discharge to a SNF. 

### 4.3. Strength and Limitations

Our study is strengthened by its population-based catchment area and the use of a uniform surgical protocol, with an established postsurgical follow-up plan. Compared with other tertiary referral heart centers, our patient population was relatively diverse, with a high percentage of black patients. Because the majority of participants lived in a primarily rural area, had similar socioeconomic status (SES), and received their care from a single regional healthcare system, this partially controlled for other healthcare-related factors that were not available in our database. 

Certain limitations should be considered when interpreting our results. Our findings from a single tertiary referral center may not be comparable to other populations undergoing TAVR. While the transfemoral access site is the most common approach for TAVR, some lower-volume hospitals in previous studies have reported a higher use of the transapical method, compared with higher-volume hospitals [37]. The latter approach has been associated with increased bleeding complications and discharge to a SNF [37,38]. Most of the TAVR procedures at our facility were performed under heavy sedation using general anesthesia. In contrast, local anesthesia may have some benefits such as increased haemodynamic stability, shorter ICU/hospital stays, and fewer postprocedural complications, which may be associated with discharge disposition [39,40,41]. This may have limited the ability to directly compare our results with other institutions that follow different protocols for sedation and TAVR access site. 

The simultaneous adjustment for two or more interrelated variables within a factor may result in misleading effect estimates [42,43]. For example, older females may be discharged to a SNF because they are more likely to be living alone. A home that is not suitable for home oxygen use may precipitate the discharge to a SNF. Low scores on the 5MWT may lead to SNF placement out of fear of falling when they return home. While such factors are likely to be representative of patients who are unable to return to their presurgical home owing to the lack of support and facilities, we were careful to avoid over adjusting for variables within a factor if they were interrelated with discharge status, as illustrated above (e.g., older age and living alone; home oxygen use and home conditions not suitable for oxygen administration; low scores on 5MWT and fear of falling). Similarly, preoperative comorbid conditions may increase the risk of downstream surgical complications. As a means to offset potential causal pathway issues, we only considered preoperative characteristics in our models. However, because of this restriction, we were unable to gauge the role of surgical outcome factors on the probability of being discharged to a SNF. This also prevented being able to analyze the mediating effect of preoperative variables on surgical outcome factors.

Our facility provides care to a higher percentage of black patients than other comparably sized centers providing specialized cardiovascular surgical services. Historically, this group has experienced differences in access to care and discrimination [44,45]. Our results also are not applicable to an increasingly younger (intermediate risk) population of patients undergoing TAVR [46]. 

A larger study including multicentric data would add to the validity of our model and may lead to the identification of other independent prognostic factors. However, the costs and practical logistics of linking ACC TVT registry data with electronic medical records at multiple sites, and associated data clean-up, was beyond the scope of the current study. Nonetheless, our results provide preliminary evidence to support a future analysis of the full ACC TVT registry. 

It also is important to recognize that TAVR has been a technologically evolving procedure and likely will continue to improve over the course of time [47]. One such advancement is the use of smaller sheath sizes, which was not considered in our analysis. As the ACC TVT registry grows in size, more precise models will be developed to optimally select patients who may benefit from this procedure, and to identify those at the greatest risk of being discharged to a SNF. Consequently, it is difficult to compare the current results with studies in the past and prudence is warranted in future applications of our model. Our results also must be carefully considered in the context of survival bias, as some patients may died before being discharged to a SNF. However, in the case of TAVR, this number is typically very small. 

There may be positive attributes of being discharged to a SNF, especially if a patient lives alone and requires more therapy. However, our study did not collect information on the living arrangements of patients, such as residential aged care and the use of home and community services. 

Finally, we acknowledge that our model may be limited by unexplained variation, residual confounding, and chance, given the observational nature of the study. We also cannot rule out that differences in assessment and reporting across clinicians may have affected our findings. Nonetheless, our risk stratification matrix is intuitive and convenient to use, as it is based on readily available information. To our knowledge, our manuscript is the first to identify a parsimonious set of preoperative risk factors and to develop a risk-stratification matrix associated with discharge to SNF following TAVR. 

### 4.4. Future Directions

In general, discharge to a SNF increases the likelihood that patient will never return home [48]. The identification of factors associated with discharge to a SNF are relevant to improving care processes and symptomatic relief of at-risk TAVR patients. Future research is merited to better understand performance measures of hospital care (e.g., functional capacity, physical and mental health, and well-being) and to appropriately apply such metrics in the context of discharge status [14,28]. Optimizing the efficiency and effectiveness of discharge planning also remains an important strategy for patients undergoing TAVR [49].

## 5. Conclusions

The risk-stratification matrix developed in the present study provides a simple tool for identifying which patients are most likely to be discharged to a SNF. Our findings are useful for clinicians and policy-makers to efficiently manage the treatment and care of patients undergoing TAVR.

## Figures and Tables

**Table 1:** Patient characteristics by discharge location, following TAVR (*N* = 285).

Characteristic	Discharge Location		*p*-Value *
	SNF n (%) Median {IQR}	${\bar{\mathrm{SNF}}}^{†}$ n (%) Median {IQR}	
Overall (n)	77	208	
Demographics			
Age (y)	83 {7}	81 {10}	0.0026
Female	48 (62)	80 (36)	0.0005
White	62 (81)	177 (85)	0.38
Assessment			
AF/Flutter	33 (43)	70 (34)	0.17
Cardiogenic shock (w/in 24 h)	3 (4)	2 (1)	0.12
Carotid stenosis	29 (38)	51 (25)	0.037
CLD (-)	46 (60)	82 (39)	0.0030
Current dialysis	5 (6)	11 (5)	0.77
Current/Recent smoker	73 (95)	184 (88)	0.12
Diabetes	41 (53)	90 (43)	0.14
5MWT (s) (pre-TAVR)	8 {6}	6 {3}	<0.0001
HF (w/in 2 w)	1 (1)	1 (<1)	0.47
Home O_2_ (-)	74 (96)	180 (87)	0.019
HTN	73 (95)	187 (90)	0.24
KCCQ-12 (pre-TAVR summary score)	37 {26}	36 {26}	0.81
NYHA (class 3–4)	66 (86)	173 (83)	0.72
Permanent pacemaker	11 (14)	20 (10)	0.29
Porcelain aorta	75 (97)	200 (96)	1.0
Prior AV procedure	11 (14)	27 (13)	0.84
Prior CABG	61 (79)	143 (69)	0.10
Prior CVA	18 (23)	33 (16)	0.16
Prior MI	33 (43)	83 (40)	0.68
Prior PCI	30 (39)	79 (38)	0.89
Clinical			
Aortic insufficiency	59 (77)	161 (77)	0.88
AV peak velocity (m/s)	4 {1}	4 {1}	0.53
CPB	11 (14)	11 (5)	0.023
Diseased vessels (-)	28 (36)	61 (29)	0.25
Non-elective procedure	21 (27)	35 (17)	0.064
MV disease	72 (94)	184 (88)	0.27
Tricuspid insufficiency	57 (74)	139 (67)	0.31

^†^ Presurgical. * Fisher’s exact test (categorical variables) or Deuchler-Wilcoxon test (continuous variables). AF = Atrial fibrillation. AV = Aortic valve. CLD = Chronic lung disease. CPB = Cardiopulmonary bypass. CVA = Cerebrovascular accident. CABG = Coronary artery bypass. 5MWT = 5-meter walk test (preoperative gait speed over 5 m). h = Hours. HF = Heart failure HTN = Hypertension. IQR = Interquartile range. KCCQ-12 = Kansas City cardiomyopathy questionnaire (12 items). m = Meters. (-) = Negative (Not present/Not receiving). MI = Myocardial infarction. MV = Mitral valve. NYHA = New York Heart Association. O_2_ = Oxygen. PCI = Percutaneous coronary intervention. s = Seconds. SNF = Skilled nursing facility. TAVR = Transcatheter aortic valve replacement. w = Weeks. y = Years.

**Table 2:** Multivariable risk model for discharge to a skilled nursing facility following TAVR (*N* = 285).

Characteristic *	RR (95% CI)	*p*-Value ^†^
Age > 75 years	2.3 (1.2–4.5)	0.0026
Female	1.6 (1.1–2.3)	0.019
5MWT > 7 s ^‡^	2.0 (1.4–3.0)	0.0002
Home oxygen (-)	2.9 (1.01–8.5)	0.0084

* Variable included in model if *p* < 0.025 and RR > 1.5 (or <0.67) in univariable analysis. **^†^** Likelihood ratio test (log-binomial regression model). ^‡^ Cutoff point based on median value. 5MWT = 5-meter walk test (preoperative gait speed over 5 m). CI = Confidence interval (Wald method). (-) = Negative (Not receiving). RR = Relative risk. s = Seconds. TAVR = Transcatheter aortic valve replacement.

**Table 3:** Risk stratification matrix for TAVR procedure (*N* = 285).

Characteristic				Probability of Discharge to SNF *	95% Confidence Interval ^†^
Age	Sex	5MWT	Home Oxygen		
>75 years	Female	>7 s	Negative (-)	0.59	0.54–0.65
			Positive (+)	0.20	0.16–0.25
		≤7 s	Negative (-)	0.29	0.24–0.35
			Positive (+)	0.10	0.07–0.14
>75 years	Male	>7 s	Negative (-)	0.39	0.32–0.44
			Positive (+)	0.13	0.09–0.17
		≤7 s	Negative (-)	0.19	0.14–0.23
			Positive (+)	0.06	0.04–0.10
≤75 years	Female	>7 s	Negative (-)	0.26	0.21–0.31
			Positive (+)	0.09	0.06–0.12
		≤7 s	Negative (-)	0.13	0.09–0.17
			Positive (+)	0.04	0.02–0.07
≤75 years	Male	>7 s	Negative (-)	0.16	0.12–0.21
			Positive (+)	0.06	0.03–0.09
		≤7 s	Negative (-)	0.08	0.05–0.11
			Positive (+)	0.03	0.01–0.05

* Log-binomial regression model. ^†^ Arcsine method. 5MWT = 5-m walk test (preoperative gait speed over 5 m). s = Seconds. TAVR = Transcatheter aortic valve replacement.
